# Alcohol use, pregnancy and associated risk factors: a pilot cross-sectional study of pregnant women attending prenatal care in an urban city

**DOI:** 10.1186/s12884-019-2652-5

**Published:** 2019-12-05

**Authors:** Imelda K. Moise

**Affiliations:** Department of Geography and Regional Studies, University of Miami, 1300 Campo Sano Ave, Coral Gables, FL 33124 USA

**Keywords:** Brief alcohol intervention, Prevalence, FASD, FAS, Substance use, Zambia, Africa, Sub-Saharan Africa, T-ACE

## Abstract

**Background:**

Alcohol consumption during pregnancy is associated with adverse pregnancy outcomes such as preventable alcohol-related developmental disability fetal alcohol syndrome. In Zambia, alcohol use and associated risk factors have not been investigated, and screening in prenatal care is nonexistent. This study determined individual correlates and the prevalence of alcohol use in pregnant women attending prenatal care at two health clinics in Lusaka, Zambia.

**Methods:**

A study adopted a cross-sectional design and recruited 188 pregnant women after seeking their informed consent from July 19 to 31, 2017. Participants aged 18 or over completed the T-ACE (Tolerance, Annoyance, Cut Down and Eye Opener) screening tool and validated alcohol-screening questionnaires on self-reported alcohol use periconceptional and during conception period while at their regular prenatal visit. The T-ACE screening tool assessed the risk of alcohol dependence in four short questions. The questionnaires included demographic questions. Bivariate analyses were performed using the χ2 test for dichotomous variables and the t-test for continuous variables. Mixed-effects linear models were used to evaluate the effect of outcome variables with patient-level variables.

**Results:**

About 40 (21.2%) pregnant women were identified by the T-ACE as at-risk for problem drinking during pregnancy. Except for regular prenatal care and distance, there was no difference in the demographic factors between pregnant women who scored < 2 on the T-ACE and those that scored > 2 points (all p’s > 0.05). A small proportional of women at both clinics reported binge drinking during the periconceptional period (12.7% vs. 3.2%, *p* = 0.003) and beyond periconception period. Excluding employed women, no significant relationships were observed between alcohol use and demographic factors.

**Conclusion:**

Alcohol consumption is prevalent in the periconceptional period and during pregnancy in pregnant women attending prenatal care in Zambia. Findings underscore the need for targeted alcohol use screening and intervention for pregnant women.

## Background

Alcohol consumption during pregnancy is a major public health problem linked to adverse pregnancy outcomes such as preventable alcohol-related developmental disability fetal alcohol syndrome (FAS) [1, 2]. It is estimated that globally around 9.8% women consume alcohol during pregnancy, with about 14.6 per 10,000 people estimated to be affected by FAS [3]. In Zambia, it is estimated that 49.3% of the population above age 15 indulge in heavy drinking (five or more drinks) on at least one occasion in the past 30 days (60.1% for men and 24.8% for women) [4], and problem drinking is greater among teen girls than teen boys [5, 6]. Studies conducted in Zambia and the Republic of South Africa (RSA) found misperceptions about alcohol use during pregnancy [7, 8].

Alcohol use during pregnancy has been found to be correlated with many negative health outcomes for the neonate (e.g., physical and cognitive defects [9] and neurodevelopmental abnormalities) [10], and for the mother (e.g., decreased production of breast milk) [11, 12]. Despite this and evidence to suggest that screening in itself can reduce alcohol consumption, rarely is alcohol screening in widespread use in prenatal care settings [13–15]. In addition, a recent review of the current situation in less developed countries found that service systems for the treatment of alcohol use disorders, where available, focus on providing tertiary care services for the treatment of dependence, often with poor outcomes [16], and are designed for men, and Zambia is no exception. Standard tests for excessive alcohol consumption include brief alcohol intervention, motivational interviewing, public awareness campaigns and the provision of additional treatment to those who screen in need of additional services in prenatal care is established [17, 18].

A vast literature in particular that conducted in RSA has shown age at onset, tobacco use, partner violence, urban living, current use and having a male partner or extended family member who drinks alcohol [19, 20] and depression [21] as risk factors for alcohol use during pregnancy. Protective factors of alcohol use while pregnant include lower gravidity and parity, education and income. These studies combined demonstrate the need for early detection strategies for prevention of alcohol use before and during pregnancy. However, in Zambia, alcohol use and associated risk factors have not been investigated, and screening in prenatal care is nonexistent; therefore, a pilot study was necessary to assess feasibility of completing alcohol screening among pregnant women within the Zambian context. The primary aim of this pilot study was to assess the feasibility of validated alcohol-screening tools in an urban Zambian city in preparation for a full-country study. Since there is lack of screening questionnaires and brief interventions developed for populations in Sub-Saharan Africa, validated questionnaires were used to determine, based on the results of this pilot, whether these questionnaire are most suitable for use in a subsequently planned national study [22]. A second objective was to assess the prevalence of alcohol consumption before and during pregnancy for purposes of calculating sample size for the full survey, and to identify and mitigate logistical challenges. The results of the first objective will be published in a separate paper.

## Methods

### Design

A cross-sectional facility-based survey was conducted at two public health clinics of Lusaka, Zambia in July 2017 about alcohol consumption before and during pregnancy by administering, validated screening questionnaires to identify problem drinking in a pregnant population (see Additional file 1) [23, 24].

### Setting

The study was located at two public primary care prenatal clinics within peri-urban and urban communities in Lusaka province Zambia where the majority face high levels of socioeconomic adversity and drug abuse. For example, it has recently been estimated that about 16.8% of the female population in Kalingalinga consume alcohol [25]. In 2017, Mtendere clinic’s catchment population was 114,064, with an annual antenatal target of 6159 while Kalingalinga clinic’s catchment population was 94,649, with an annual antenatal target of 5111.

### Participants

A sample of pregnant women were recruited from two public health clinics of Kalingalinga urban clinic and Mtendere peri-urban clinic, which provide free prenatal care to pregnant women in these parts of Lusaka province in Zambia. At Kalingalinga urban clinic, 90 women were approached and 79 (87.78%) completed the tool vs. 109 of 120 women approached at Mtendere clinic (90.83%). To be eligible to complete a T-ACE (Tolerance (T); Annoyed (A); Cut down and eye-opener (E)) screening tool, women had to be pregnant, aged 18 or over and visiting one of the clinics at the time of the study. Excluded from the study were eligible women who refused to answer the T-ACE tool or the other study questionnaires (*n* = 8 at Kalingalinga clinic and *n* = 6 at Mtendere clinic) and adolescents aged below 16.

Every third woman attending their first prenatal visit was invited to participate in the study through systematic sampling as previously used in similar studies [26]. A major advantage of this approach is that it optimized inclusion of women who were willing to participate, maximized the effectiveness of data collection efforts, and because the consideration of subjects representative of the entire population was not an objective of this study [27].

Recruitment occurred from July 19 to 31, 2017. Women were recruited with the help of the district health manager. Researchers approached pregnant women while they were waiting for their routine prenatal care, provided at set times each day of the week, and to reach the maximum number of women, recruitment occurred during these times. Those who opted to participate in the study received verbal and written information about the study and reassurance about confidentiality. If they agreed to participate, they completed the alcohol-screening questionnaire right away in English or in a preferred participant local language of choice (Bemba and Nyanja). The questionnaire was completed in private and non-judgmental settings (e.g., conference room).

### Data collection

All women who provided written consent to participate were screened in two stages. First, administered was a brief sociodemographic questionnaire. Demographic information included age, gender, and socioeconomic status as approximated by parental education. Second was verbal and self- administration of two commonly used alcohol-screening questionnaires in a face-to-face interview conducted by the researcher and a trained research assistant.

### Measures

A validated T-ACE alcohol-screening questionnaire was used based on its widespread use in detecting tolerance to alcohol and lifetime alcohol abuse or addiction issues. The tool was translated in Bemba and Nyanja, two languages commonly spoken in Lusaka by the author (native speaker), and validated in these languages. The T-ACE has been found to have high levels of sensitivity (69–88%) and specificity (71–89%) for identifying risk drinking and problem drinking among pregnant women [13], and has been validated for use in wide range of settings [17, 28, 29] including Sub-Saharan Africa [30], and for verbal administration by an interviewer in both English and local language. A score of two or more on the “T-ACE” indicates at-risk drinking [23]. Four questions comprise the T-ACE: [1] How many drinks does it take to make you feel high? [2] Have people annoyed you by criticizing you’re drinking? [3] Have you ever felt you ought to cut down on your drinking? [4] Have you ever had a drink first thing in the morning to steady your nerves or get rid of a hangover?

To identify overall severity of dependence or periconceptional period alcohol use, five-item questions were administered along with the T-ACE screening tool: [1] During the time you were pregnant, but didn’t know you were pregnant, how many alcohol drinks did you usually have at one time? [2] During the time you were pregnant, but didn’t know you were pregnant, how often did you drink beer, wine or other alcoholic beverages? [3] How often did you have four or more drinks in one day in the past 30 days? [4] How many drinks did you have on a typical day when you were drinking alcohol in the past 30 days? [5] During the past 30 days, on how many days did you drink one or more drinks of an alcoholic beverage?

### Analysis

IBM SPSS Statistics, version 24.0 (Armonk, NY: IBM Corp) was used to perform descriptive statistics. To compare demographic factors and alcohol use patterns among participants by clinic and among those who scored two or more points on the T-ACE questionnaire, bivariate analyses were performed using the χ^2^ test for dichotomous variables and the t-test for continuous variables. Because alcohol use data were not normally distributed, I used medians and first (Q1) and third (Q3) quartiles to describe the data distribution on any alcohol use and binge drinking episode in the past 30 days (number of drinks and drank ≥4 alcoholic drinks on at least one day), and any at-risk drinking (scored > 2 on the T-ACE questionnaire). A mixed-effects linear model (188 pregnant women in 10 wards) was used to evaluate the effect of the log-transformed outcome variables (number of drinks consumed ≥4 alcoholic drinks on at least one day in the past 30 days and scoring > 2 on the T-ACE questionnaire), after adjusting for patient-level variables. Patient-level variables included age, marital status, education and prenatal care regular attendance. The mixed-effects analysis was used to account for dependence resulting from pregnant women being nested within administrative wards. Statistical significance of fixed effects was evaluated at *p* < .05.

## Results

### Participants

The mean age of participants at the time of screening was 26.8 (range: 14–41) years and mean gestational age was 24.4 (range: 4–42; median = 24.0; Quartile 1 = 16; Quartile 3 = 31.5) weeks. The self-reported mean age at first use of alcohol was 19.9. Only 9.0% of screened participants were primigravida and the majority were unemployed (69.1%).

### At risk drinking during pregnancy

Of all participants, about 40 (21.2%) were identified by the T-ACE as at-risk for problem drinking during pregnancy. Except for 2 of 12 variables used, no differences in the demographic factors were detected between participants who scored < 2 points and those that scored > 2 points on the T-ACE (all *p*’s > 0.05) (Table 1). Regular prenatal use was lower among participants who scored > 2 points compared to those who scored < 2 points on T-ACE (48.8% vs. 20.8%, *p* < 0.001). A higher proportion of participants who scored < 2 points on T-ACE did not perceive distance as a constraint to prenatal care access (26.2% vs. 9.5%; *p* < 0.001).

**Table 1:** Demographic characteristics of screened pregnant women by T-ACE scores: Lusaka, Zambia, July 2017 (*n* = 188)

	T-ACE Scored		
	< 2 points (*n* = 116)	> 2 points (*n* = 40)	
	Mean (95% CI)	Mean (95% CI)	*p*-value
Maternal age at screening (years)	27.0 (25.9–28.1)	25.7 (24.0–27.4)	0.299
Gestational age at screening (weeks)	24.2 (22.6–25.9)	24.5 (21.7–27.2)	0.808
Number of times pregnant	3.0 (2.8–3.2)	2.9 (2.51–3.19)	0.086
Maternal age first time had alcohol drink	18.4 (17.2–19.6)	19.6 (17.7–21.4)	0.296
	N (%)	N (%)	
Employment status			0.602
Unemployed	68 (40.5)	25 (14.9)	
Employed	16 (9.5)	9 (5.4)	
Marital status			0.934
Single (never married/dating)	15 (8.9)	7 (4.2)	
Married	82 (48.85)	30 (17.9)	
Divorced/separated/windowed	3 (1.8)	1 (0.6)	
Education			0.432
Some primary school	11 (6.5)	7 (4.2)	
Primary school	24 (14.3)	9 (5.4)	
Secondary school	48 (28.6)	18 (10.7)	
> college	17 (10.1)	3 (1.8)	
Religion			0.756
Christian (e.g., Catholic, protestant)	98 (58.3)	38 (22.6)	
Muslim	0 (0.0)	0 (0.0)	
Not religious	1 (0.6)	0 (0.0)	
Household income (monthly)			0.569
Dependent (no income)	31 (18.5)	11 (6.5)	
≤ 1000 ZMW	15 (8.9)	8 (4.8)	
> 1000 ZMW	37 (22.0)	12 (7.1)	
Primary source of emotional support			0.580
Parent or relative	42 (25.0)	20 (11.9)	
Spouse or significant other	51 (30.4)	15 (8.9)	
Friend	6 (3.6)	1 (0.6)	
Prenatal care (regular)			0.001
no	17 (10.1)	2 (1.2)	
yes	82 (48.8)	35 (20.8)	
Distance factor to accessing prenatal care			0.001
no	44 (26.2)	16 (9.5)	
yes	30 (17.9)	12 (7.1)	

**Note**: Descriptive statistics were calculated and reported for women who answered the questions as means with associated confidence intervals, and percentages as appropriate. Only participants who responded to all the four T-ACE questions are included in the analysis (*n* = 156 or 188 participants)

T-ACE Questionnaire: T, how many drinks does it take to make you feel high? A, Annoyed, Have people annoyed; you by criticizing your drinking?; C, Cut down, Have you ever felt you ought to cut down on your drinking); and.

E, Eye-opener asked, have you ever had a drink first thing in the morning to steady your nerves or get rid of a hangover?

### At risk drinking during the Periconceptional period

While most participants in the sample (66%, *n* = 124) reported never drinking any alcohol, about 12.7% in the Kalingalinga and 3.2% at Mtendere samples, *p* = 0.003 reported binge drinking (consuming ≥4 drinks) during the periconceptional period (Table 2). A somewhat smaller proportion of participants reported binge drinking beyond periconceptional period (representing 2^nd^ and 3^rd^ trimesters for most participants) that is consuming ≥4 drinks in at least one day during the past 30 days, and scoring > 2 or more drinks on the T-ACE (9.0% vs. 8.0% at Kalingalinga and 16.0% vs. 13.3% at Mtendere).

**Table 2:** Alcohol use among screened pregnant women by clinic, Lusaka, Zambia, July 2017 (*n* = 188)

	Clinics		
	Kalingalinga (*n* = 79)	Mtendere (*n* = 109)	
	n (%)	n (%)	*p*-value
Alcohol use in the periconceptional period^a^			0.003
Did not consume any alcohol	52 (27.7)	22 (31.9)	
Consumed alcohol (≤4 drinks at one time)	2 (1.1)	21 (11.2)	
Any binge drinking^a^	10 (12.7)	6 (3.2)	
Frequency of drinking			0.557
Everyday	0 (0.0)	4 (2.1)	
3–4 days a week	1 (0.5)	2 (1.1)	
1–2 days a week	3 (1.6)	6 (3.2)	
2–3 days a week	0 (0.0)	0 (0.0)	
Once a month	4 (2.1)	8 (4.3)	
Less than once a month	1 (0.5)	4 (2.1)	
Never (don’t drink)	57 (30.3)	67 (35.6)	
Alcohol use in the past 30 days			
Consumed no alcoholic drinks in at least one day	50 (26.6)	57 (30.3)	0.322
Any binge drinking at least one day/weekly average	17 (9.0)	30 (16.0)	
T-ACE alcohol-screening questionnaire			0.613
Scored < 2 or more points	52 (27.7)	64 (34.0)	
Scored > 2 or more points (risk drinking during pregnancy) ^b^	15 (8.0)	25 (13.3)	

^a ≥^4 drinks at one time during the time the woman was pregnant but did not know she was pregnant;

^b^Based on the T-ACE standard cut-off point-- responding “2 or more drinks” on the T-ACE question # 1

### Demographic associations with past 30 days alcohol use

Adjusted multivariable analyses further indicated that the odds of at-risk drinking during the past 30 days was 1.002 more likely among employed participants than it was for unemployed participants (95% CI, 0.1.000–1.003). There were no significant relationships between at risk drinking during the past 30 days and other neighborhood demographics; and between participants who scored ≥2 on the T-ACE and demographics factors (all p’s > 0.05; data not shown).

## Discussion

The current study found that alcohol consumption is prevalent in the periconceptional period and during pregnancy in participants attending prenatal care at the two study clinics in Zambia. To note, although most participants in the sample reported never consuming any alcohol in the periconceptional period and during pregnancy, almost a quarter of participants at both clinics were identified as at-risk for problem drinking during the periconceptional period and during pregnancy. Prevalence estimates obtained in this study are alarming, particularly given the self-reported nature of the study, small sample size and because there is no safe amount of alcohol to drink while pregnant. Therefore, while these results do not imply cause and effect, these findings suggest that Zambian women who consume any alcohol during pregnancy may be at increased risk for poor pregnancy outcomes [31] due to lack of alcohol screening in prenatal care.

Protective factors for at risk drinking during pregnancy in Zambia include attending prenatal care regularly and proximity to antenatal care clinics (measured here as distance to prenatal services), suggesting that improving structural constraints (e.g., improved transportation) may improve access and utilization of antenatal care and the behavior of pregnant women. This finding broadly supports the work of other studies in this area linking actual travel distance with access to reproductive health services [32, 33]. Further analysis also revealed that the odds of risk drinking during the past 30 days was higher among employed pregnant women compared to unemployed pregnant women.

Potential limitations to this study include reliance on participant self-reporting of alcohol use before and during pregnancy. In addition, given the stigmatization associated with alcohol consumption during pregnancy, the prevalence estimates, while high, might be an underestimation. Further, some participants could not accurately recall their previous alcohol use. In addition, the study was cross sectional, precluding discussion of temporality nor can causality be inferred. The study only included maternal factors based on literature and other factors that may relate to alcohol use during pregnancy were not included in the analysis. Nevertheless, the findings support increased efforts to develop and implement evidence-based interventions to prevent and reduce alcohol use during pregnancy.

## Conclusions and recommendations

Understanding barriers, risks, and protective factors associated with at risky drinking in pregnancy, early screening, counselling including the provision of additional treatment to those who screen in need of additional services, and other factors (e.g., personal and environmental) may be useful in the development of policies and interventions aimed at screening and reducing drinking during pregnancy and related consequences.

## Supplementary information

**Additional file 1.** Alcohol Brief Intervention First Visit Screening Questions with T-ACE.

## Data Availability

Datasets generated during the current study and for this manuscript are available from the corresponding author on reasonable request.

## Abbreviations

CI: Confidence Interval
FAS: Fetal Alcohol Syndrome
FASD: Fetal Alcohol Spectrum Disorder
RSA: Republic of South Africa
T-ACE: Tolerance-Annoyance, Cut Down, Eye Opener
