# Supplementary material for: Alcohol use, pregnancy and associated risk factors: a pilot cross-sectional study of pregnant women attending prenatal care in an urban city
Source: BMC Pregnancy Childbirth. 2019 Dec 5;19:472. doi: 10.1186/s12884-019-2652-5 (PMC6896278; doi:10.1186/s12884-019-2652-5)
Supplement: Supplementary file 1 — Additional file 1. Alcohol Brief Intervention First Visit Screening Questions with T-ACE. [file 12884_2019_2652_MOESM1_ESM.doc]

**Alcohol Brief Intervention First Visit Screening Questions with T-ACE**

**Participant ID____________________________________**

**Date of Visit:** __ __/__ __/__ __ __ __

Mo Day Yr

**1. How many weeks pregnant are you today?** ________weeks

Use the standard drink chart (last page) to answer Questions 2–6. (One standard drink is equal to 12 ounces of beer, 5 ounces of wine, or 1.5 ounces [one shot] of 80-proof spirits or liquor.)

**2. During the time you were pregnant but didn’t know you were pregnant, how many alcoholic drinks**

**did you usually have at one time?** Circle your answer.

10 or more 9 8 7 6 5 4 3 2 1 0

**3. During the time you were pregnant but didn’t know you were pregnant, how often did you drink**

**beer, wine, or other alcoholic beverage?** Check a box for your answer.

🞏 Every day

🞏 Almost every day

🞏 3-4 days a week

🞏 1-2 days a week

🞏 2-3 days a month

🞏 Once a month

🞏 Less than once a month

🞏 Never

**4. How often did you have 4 or more drinks in one day in the past 30 days?** Circle your answer.

10 or more 9 8 7 6 5 4 3 2 1 0

**5. How many drinks did you have on a typical day when you were drinking alcohol in the past 30 days?** Circle your answer.

10 or more 9 8 7 6 5 4 3 2 1 0

**6. During the past 30 days, on how many days did you drink one or more drinks of an alcoholic**

**beverage?**

Write one number between 0 and 30 days as your answer: *________*

| **This may help you estimate the number of days you drank:** |
| --- |
| Drinking every day would be 30 days. |
| Almost every day would be a number between 17 to 29 days. |
| 3–4 days a week would be a number between 12 to 16 days. |
| 1–2 days a week would be a number between 4 to 8 days. |
| 2–3 days a month would be either 2 or 3 days. |
| Once a month would be 1 day. |
| Never would be 0 days. |

Circle your answers to the questions below.

1. **How many drinks does it take to make you feel high?**

10 or more 9 8 7 6 5 4 3 2 1

**2. Have people annoyed you by criticizing your drinking?** No Yes

**3. Have you ever felt you ought to cut down on your drinking?** No Yes

**4. Have you ever had a drink first thing in the morning to steady**

**your nerves or get rid of a hangover?** No Yes
